# The role of segmental nodes in the pathological staging of non-small cell lung cancer

**DOI:** 10.1186/1749-8090-8-225

**Published:** 2013-12-08

**Authors:** Zhen-xuan Li, Hong Yang, Ke-lin She, Ming-xing Zhang, Han-qing Xie, Peng Lin, Lan-jun Zhang, Xiao-dong Li

**Affiliations:** 1 State Key Laboratory of Oncology in South China, Sun Yat-sen University Cancer Center, 651 Dongfeng Rd, East, Guangzhou, PR China; 2 Cancer Hospital of Henan province, Zhengzhou, PR China; 3 Central Hospital, Shaoyang, PR China

**Keywords:** Non-small cell lung cancer, Lymph node, Segmental node, Pathological staging, Radical resection

## Abstract

**Background:**

Segmental nodes are not examined routinely in current clinical practice for lung cancer, the role of segmental nodes in pathological staging of non-small cell lung cancer after radical resection was investigated.

**Methods:**

A total of 113 consecutive non-small cell lung cancer patients who underwent radical resection between June 2009 and December 2011 were retrospectively reviewed. All the operations were performed by the same group of surgeons. N2 nodes, hilar nodes, interlobar nodes and some lobar nodes were collected during surgery. The removed lung lobes were dissected routinely along lobar and segmental bronchi to collect lobar nodes and segmental nodes. The collected lymph nodes were separately labeled for histological examination.

**Results:**

The detection rates of hilar nodes, interlobar nodes, lobar nodes and segmental nodes were 61.1%, 85.0%, 75.2% and 80.5%, respectively. The metastasis rates of hilar nodes, interlobar nodes, lobar nodes and segmental nodes were 5.3%, 10.5%, 16.8% and 14.2%, respectively. There were 68 cases of N0 disease, 16 cases of N1 disease and 29 cases of N2 disease. If an analysis of segmental lymph nodes had been omitted, six patients (37.5% of N1 disease) would have been down-staged to N0, and two cases of multiple-zone N1 disease would have been misdiagnosed as single-zone N1 disease, one patient would have been misdiagnosed as N2 disease with skip metastases.

**Conclusion:**

Segmental nodes play an important role in the accurate staging of non-small cell lung cancer, and routinely dissecting the segmental bronchi to collect the lymph nodes is feasible and may be necessary.

## Background

Accurate pathological staging of lymph node involvement has been recognized as a key factor in the management of lung cancer, which includes the postsurgical treatment selection and prognosis prediction. The 5-year survival rates of N0, single-zone N1, multiple-zone N1 and N2 disease for surgically managed patients with non-small cell lung cancer (NSCLC) are 56%, 48%, 35% and 22%, respectively [1]. Based on the current staging, adjuvant therapy can be used to improve survival [2,3]. Referring to the International Association for the Study of Lung Cancer (IASLC) lymph node map [4], levels 10–12 lymph nodes (hilar nodes, interlobar nodes and lobar nodes) are examined routinely in current clinical practice, but level 13 lymph nodes (segmental nodes) are not. The role of segmental nodes in the pathological staging of lung cancer has rarely been reported. Accordingly, we retrospectively analyzed the influence of segmental nodes for patients with non-small cell lung cancer undergoing radical resections, which were performed by the same group of surgeons.

## Methods

This study was approved by the Sun Yat-Sen University Cancer Center Institutional Review Board of Clinical Research. The need for informed consent from patients was waived due to its retrospective design.

### Patient eligibility

Between June 2009 and December 2011, 127 consecutive patients with lung cancer underwent surgical resection in our treatment group at the Sun Yat-Sen University Cancer Center. The inclusion criteria are listed here: (1) Patients with non-small cell lung cancer who underwent pulmonary resection plus complete mediastinal lymph node dissection (MLND), (2) patients who underwent R0 (microscopically complete) resection and (3) patients who did not have distant metastasis. One hundred and thirteen patients were enrolled. All patients underwent computed tomography (CT) scans of the chest and upper abdomen with intravenous contrast, bronchoscopy, and brain magnetic resonance imaging prior to surgery. Some patients underwent positron emission computed tomography (PET), PET-CT, bone scanning or mediastinoscopy for exact clinical staging. The 7th edition of the AJCC and UICC TNM classification for lung cancer was used for staging [5].

### Surgical technique

**Figure 1:**
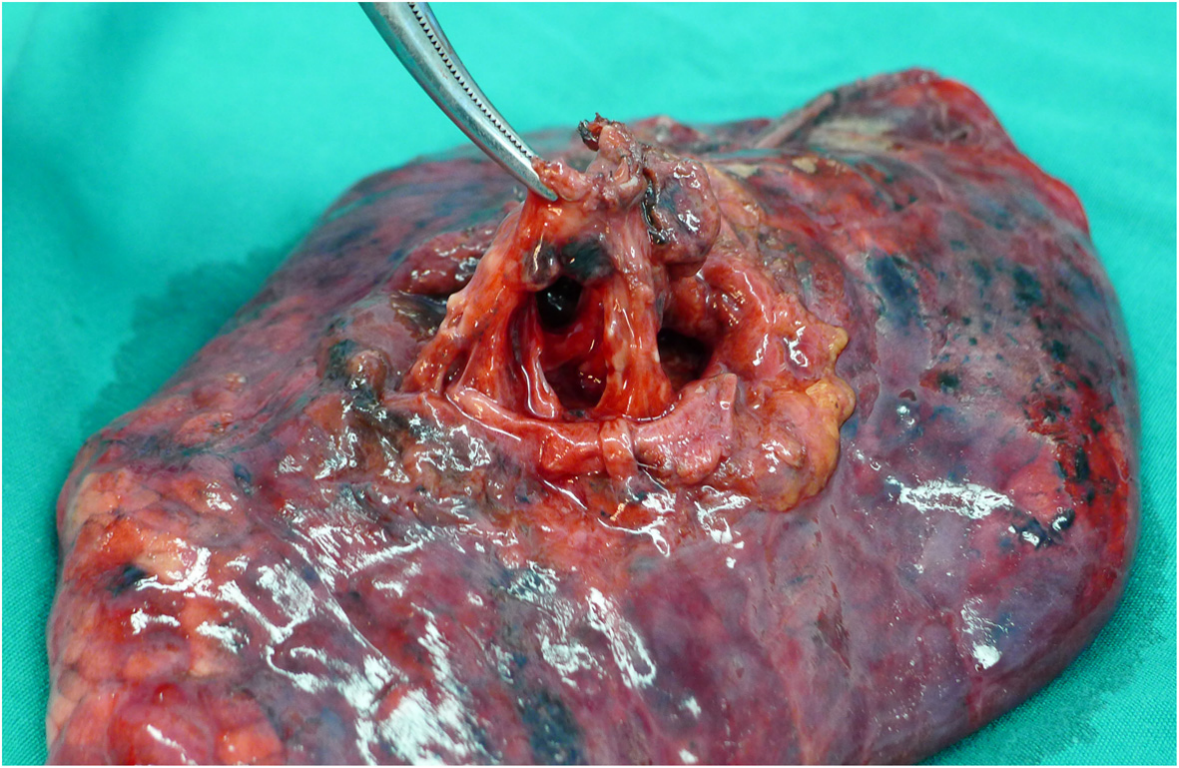
Right upper lobe.

**Figure 2:**
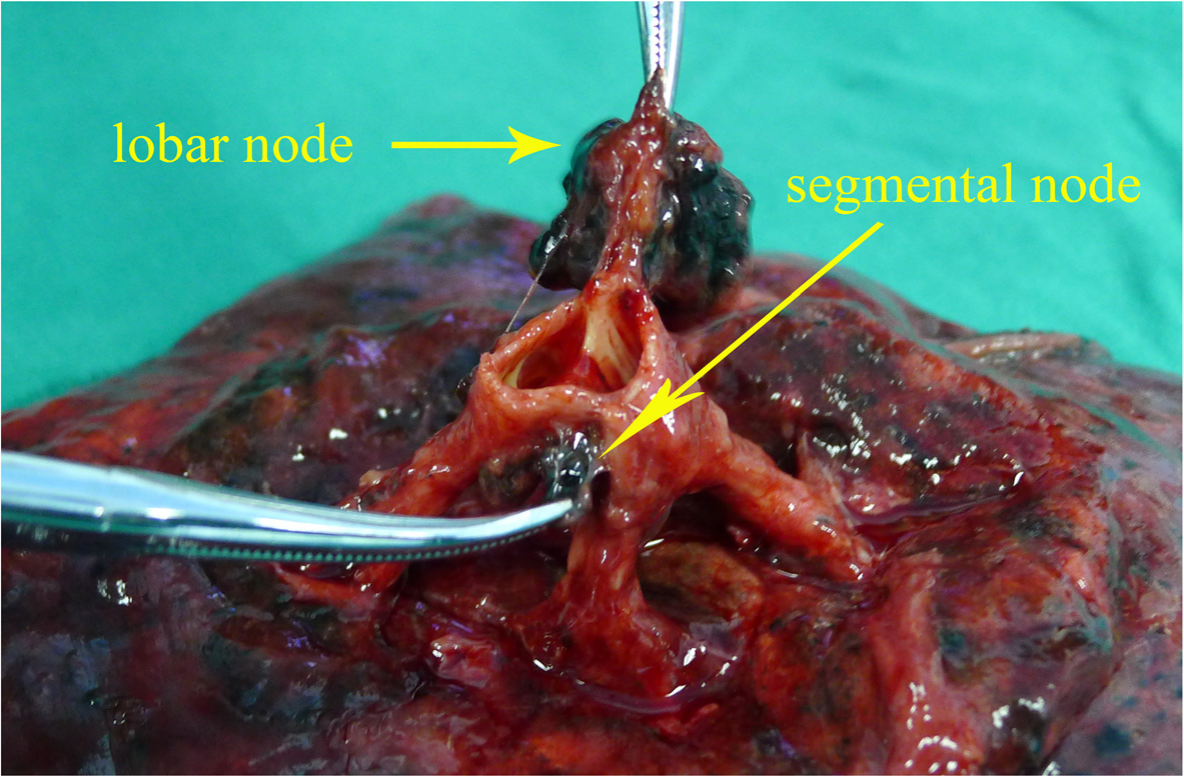
Lobar node and segmental node.

Pulmonary resection plus complete MLND by thoracotomy or video-assisted thoracoscopic surgery (VATS) was performed, and extended resection was performed in some cases to achieve R0 resection. The technique “lymph node dissection” was defined as the en bloc removal of all tissue that may contain cancer cells, including the lymph nodes and surrounding fatty tissue within anatomic landmarks, such as the trachea, bronchus, superior vena cava, aorta and its branches, pulmonary vessels, and pericardium [6]. The details of the procedure were described in our previous report [7]. Complete MLND means that levels 2–4 and 7–9 lymph nodes were en bloc removed for right lung cancer patients, and levels 4–9 lymph nodes were removed for left lung cancer patients [7]. Levels 10–12 lymph nodes were collected during the surgeries. After surgery, the pulmonary lobes that had been removed were routinely dissected by the surgeon to collect levels 12–13 lymph nodes. Segmental nodes are defined as the lymph nodes adjacent to the segmental bronchi [4], whose upper border is the origin of the segmental bronchi, and whose lower border is the origin of the subsegmental bronchi. We usually cut lung tissue with scissors along the bronchial wall and the segmental bronchial wall to gather the lobar and segmental nodes (Figures 1 and 2). The collected lymph nodes were separately labeled for histological examination.

### Statistical methodology

Numerical data were expressed as mean ± standard deviation or median, categorical data were expressed as frequencies or ratios. The chi-square or Fisher exact tests were used to analyze differences among the categorical data. All statistical tests reported in the manuscript were two-sided. Values of *p* < 0.05 were considered to be statistically significant. All statistical analyses were performed using the SPSS 13.0 for Windows (SPSS Inc, Chicago, IL).

## Results

Patient characteristics were shown in Table 1. The median age was 60.0 years (range, 33–76 years). The median tumor diameter was 3.0 cm (range, 0.5-11 cm). Thoracotomy was performed for 57 patients, while VATS was performed for 56 patients. Patients underwent lobectomy or bilobectomy (n = 102), lobectomy with a bronchial sleeve resection (n = 4) and pneumonectomy (n = 7). Four patients underwent neoadjuvant chemotherapy, of which one case achieved complete response (CR), and three cases achieved partial response (PR). One patient undergoing neoadjuvant chemotherapy had N1 disease with only segmental lymph node involvement. All patients underwent complete MLND, and no perioperative deaths occurred.

**Table 1:** Patient characteristics

**Characteristic**	**No. of patients (%) (N = 113)**
Sex	
Male	74 (65.5)
Female	39 (34.5)
The site of the primary tumor	
Right upper	37 (32.7)
Middle	5 (4.4)
Lower	25 (22.1)
Center	1 (0.9)
Left upper	23 (20.4)
lower	20 (17.7)
Center	2 (1.8)
Histology	
Squamouse cell carcinoma	34 (30.1)
Adenocarcinoma	62 (54.9)
Others	17 (15.0)
Clinical stage	
CR	1( 0.9)
IA	43 (38.1)
IB	11 (9.7)
IIA	12 (10.6)
IIB	8 (7.1)
IIIA	38 (33.6)
Pathological stage	
CR	1 (0.9)
IA	27 (23.9)
IB	22 (19.5)
IIA	16 (14.1)
IIB	13 (11.5)
IIIA	34 (30.1)

The mean numbers of N2 nodes, hilar nodes, interlobar nodes, lobar nodes and segmental nodes were 23.4 ± 10.5, 2.0 ± 2.3, 3.1 ± 2.4, 2.9 ± 2.7, and 2.2 ± 1.8, respectively. The detection rates for hilar nodes, interlobar nodes, lobar nodes and segmental nodes were 61.1%, 85.0%, 75.2% and 80.5%, respectively. The metastasis rates for hilar nodes, interlobar nodes, lobar nodes and segmental nodes were 5.3%, 10.6%, 16.8% and 14.2%, respectively (Table 2). There were 29 cases of N2 disease, 16 cases of N1 disease and 68 cases of N0 disease. One patient with a left upper-lobe tumor had metastatic lymph nodes at levels 5, 6 and 13. Eleven patients presented with skip metastases (N2 disease in the absence of N1 metastasis). Lymph node stations were grouped together into six zones by IASLC [1]: peripheral (levels 12–14) or hilar (levels 10 and 11) for N1 nodes and upper or lower mediastinal, aortopulmonary, and subcarinal for N2 nodes. We identified 3 patients with multiple-zone N1 disease and 13 patients with single-zone N1 disease (Table 3). Forty-five patients had metastatic lymph nodes, of which 35.6% (16/45) had segmental node metastasis. Among patients with N1 disease, the cases with metastatic segmental nodes accounted for 56.3% (9/16), which is a greater percentage than other N1 stations (level 10, 6.3%; level 11, 31.3%; level 12, 31.3%). If an analysis of segmental nodes had been omitted, six patients (37.5% of N1 disease) would have been down-staged to N0. Furthermore, two cases of multiple-zone N1 disease would have been misdiagnosed as single-zone N1 disease. In addition, one patient who had a left upper-lobe tumor with metastatic lymph nodes at levels 5, 6 and 13 would have been misdiagnosed with AP zone disease in the absence of N1 metastases.

**Table 2:** The detection and metastasis of N1 nodes

	**ND**	**DR**	**NM**	**MR**
Level 10	69	61.1%	6	5.3%
Level 11	96	85.0%	12	10.6%
Level 12	85	75.2%	19	16.8%
Level 13	91	80.5%	16	14.2%

*ND*, number of cases that have lymph nodes were detected; *DR*, detection rate;

*NM*, number of cases that have metastatic lymph nodes; *MR*, metastasis rate.

**Table 3:** Metastatic type of N1 disease

**Lymph node station**	**No. of patients**
Multiple-zone N1	3
Levels 13 + 11	1
Levels 13 + 10	1
Levels 12 + 11	1
Single-zone N1	13
Levels 13	6
Level 12	3
Level 11	3
Level 13 +12	1

In the subgroup of patients who had tumor diameters >3 cm, the detection rate for segmental nodes was higher than for patients who had tumor diameters ≤3 cm (50/55 vs. 41/58, P = 0.007). Patients with history of smoking had higher detection rates of segmental nodes (53/60 vs. 38/53, P = 0.026). Detection rates for segmental nodes were not significantly different, irrespective of location, histology, differentiation, and clinical staging. The metastasis rates of segmental nodes were not statistically different, regardless of tumor size, history of smoking, location, histology, differentiation and clinical staging.

## Discussion

Accurate pathological staging of lymph node involvement plays an important role in the prediction of prognosis and the selection of treatment strategies for lung cancer. Pulmonary resection plus complete MLND has proven effective in obtaining accurate N staging [8,9]. Among the previous complete MLND randomized controlled trials [8-12], Wu Y [8] and Izbicki J R [10] did not mention the details of N1 disease in their study. Passlick B [11] and Sugi K [12] did not explicitly report whether they dissected segmental nodes, and no metastatic segmental nodes in N1 disease were reported. The American College of Surgeons Oncology Group Z0030 trial [13] mentioned segmental nodes. In the Z0030 study, segmental nodes were harvested from only 11.5% (60/524) of the patients who underwent MLND. However, in our study, segmental nodes were harvested from 80.5% (91/113) of the patients. Overall, the detection of segmental nodes has generally been overlooked in current clinical practice.

In our study, level 13 had a high detection rate. It has been suggested that lymph nodes are mainly distributed at the intersection of vessels [14]. Lymphatic drainage of the lungs occurs via a superficial subpleural lymph plexus and a deep plexus of lymph vessels accompanying the bronchi. Both groups drain through the hilar or bronchopulmonary nodes to the tracheobronchial nodes and thence to the mediastinal lymph trunks [14]. Based on this, the segmental nodes could often be involved during lung cancer metastasis. In our study, level 13 has the highest metastasis rate in N1 disease, which is correspond with the lymphatic drainage accompanying the bronchi.

Moreover, level 13 could be the single station of metastatic lymph nodes in lung cancer patients, as has been demonstrated in some reports [15-18] with rates from 3.2% to 29.7%. Maeshima A M [19] reported patients with pN1 status who had only level 13/14 lymph node metastasis had an intermediate 5-year disease-free survival rate between that of patients with pN0 status and other patients with pN1 status. They supported the conclusion that routine examination of level 13/14 lymph nodes is important for accurate pathologic staging and for the prediction of clinical outcome of patients with NSCLC. In our study, 6 patients had single station of metastatic segmental nodes. If an analysis of segmental nodes had been omitted, these patients would have been down-staged to N0. We will further our study to investgate whether segmental nodes metastasis impact survival or recurrence.

N1 disease has a heterogeneous prognosis in relation to node descriptors [1,16,20-23]. The prognosis of single-zone N1 disease has proven better than multiple-zone N1 disease [1,20], and single-station N1 disease has a better prognosis than multiple-station N1 disease [20-23]. N2 disease also has a heterogeneous prognosis with regard to node descriptors. In the absence of N1 metastasis, AP zone disease was associated with better survival rates in patients with left upper-lobe tumors [1]. N2 disease with skip metastases had a more favorable prognosis than N2 disease that lacked skip metastases [24-27]. If the segmental nodes had not been examined in our study, two patients with multiple-zone N1 disease would have been misdiagnosed as single-zone N1; three cases with multiple-station N1 disease would be misjudged as single-station N1; and one patient who had a left upper-lobe tumor with metastases at levels 5, 6 and 13 would have been misdiagnosed with AP zone (levels 5, 6) disease in the absence of N1 metastases. Without examining the segmental nodes, prognosis estimations could be incorrect.

The subgroup analysis revealed that the segmental nodes were easier to detect in patients who had tumor sizes > 3 cm in diameter, possibly because the growth of the tumor stimulated the immune system. The metastasis rates of segmental nodes were not significantly different, regardless of tumor size, location, histology, differentiation and clinical staging. Thus, we cannot selectively dissect segmental nodes. There are two reasons why segmental nodes are rarely dissected in current clinical practice. First, some surgeons and pathologists do not realize the importance of the segmental nodes for the staging and treatment of lung cancer. Second, some surgeons and pathologists think that the detection of segmental nodes is difficult and time-consuming; as a result, they are reluctant to try looking for the segmental nodes. According to our experience, the segmental nodes are usually located at the intersection between the lobar bronchus and the segmental bronchi. After surgery, we cut lung tissue with scissors along the wall of the bronchi and segmental bronchi, which exposes the segmental nodes. It usually takes three minutes for us to perform the procedure, the results of which may benefit the patient throughout his or her life. Thus, we believe that routinely collecting the segmental nodes is necessary and feasible.

## Conclusions

Segmental nodes play an important role in the accurate staging of non-small cell lung cancer, and routinely dissecting the segmental bronchi to collect the lymph nodes is feasible and may be necessary.

## Abbreviations

NSCLC: Non-small cell lung cancer; IASLC: International association for the study of lung cancer; MLND: Mediastinal lymph node dissection; VATS: Video-assisted thoracoscopic surgery; ND: Number of cases that have lymph nodes were detected; DR: Detection rate; NM: Number of cases that have metastatic lymph nodes; MR: Metastasis rate.

## Competing interests

The authors declare that they have no competing interests.

## Authors’ contributions

LZX conducted the study and performed the statistical analysis and drafted the manuscript. YH conducted the study and collected the data. LXD conceived of and supervised the study. SKL, ZMX, XHX, LP and ZLJ participated in its design and coordination and helped to draft the manuscript. All authors read and approved the final manuscript.
